# Effect of Heat Stress on Some Physiological and Anatomical Characteristics of Rice (*Oryza sativa* L.) cv. KDML105 Callus and Seedling

**DOI:** 10.3390/biology11111587

**Published:** 2022-10-28

**Authors:** Worasitikulya Taratima, Chantima Chuanchumkan, Pitakpong Maneerattanarungroj, Attachai Trunjaruen, Piyada Theerakulpisut, Anoma Dongsansuk

**Affiliations:** 1 Department of Biology, Faculty of Science, Khon Kaen University, Khon Kaen 40002, Thailand; 2 Salt Tolerant Rice Research Group, Faculty of Science, Khon Kaen University, Khon Kaen 40002, Thailand; 3 Faculty of Veterinary Medicine, Khon Kaen University, Khon Kaen 40002, Thailand; 4 Department of Agronomy, Faculty of Agriculture, Khon Kaen University, Khon Kaen 40002, Thailand

**Keywords:** global warming, leaf surface anatomy, rice callus, seedling growth, Thai jasmine rice

## Abstract

**Simple Summary:**

Climate change is currently threatening agriculture all around the world, resulting in a lack of water and restricting the growth of plants, especially rice. Rice production decreases with the increase in temperature. An improvement in fundamental knowledge is necessary to comprehend plant adaptation mechanisms as responses to heat stress. Physiological and anatomical responses of Khao Dawk Mali 105 (KDML105) rice to artificial heat stress were studied. Our findings offer useful data for projects aimed at improving heat stress tolerance in rice to enhance long-term global food security.

**Abstract:**

Global warming is a serious problem, with significant negative impacts on agricultural productivity. To better understand plant anatomical adaptation mechanisms as responses to heat stress, improved basic knowledge is required. This research studied the physiological and anatomical responses of Khao Dawk Mali 105 (KDML105) to artificial heat stress. Dehusked seeds were sterilized and cultured on Murashige and Skoog (MS) medium, supplemented with 3 mg/L 2,4-Dichlorophenoxyacetic acid (2,4-D) for callus induction. The cultures were maintained at 25 °C and 35 °C for 4 weeks, while the other culture was treated with heat shock at 42 °C for 1 week before further incubation at 25 °C for 3 weeks. Results revealed that elevated temperatures (35 °C and 42 °C) adversely impacted seedling growth. Plant height, root length, leaf number per plant, fresh and dry weight, chlorophyll a, chlorophyll b and total chlorophyll content decreased after heat stress treatment, while malondialdehyde (MDA) and electrolyte leakage percentage significantly increased, compared to the control. Heat stress induced ROS accumulation, leading to lipid peroxidation and membrane instability. Principal component analysis (PCA) and hierarchical cluster analysis (HCA) results also confirmed negative correlations between MDA, electrolyte leakage and other parameters. MDA content and electrolyte leakage are effective indicators of heat stress in rice. Surface anatomical responses of rice seedlings to heat stress were studied but significant alterations were not observed, and heat stress had no significant negative effects on KDML105 calli. Size and mass of calli increased because heat stress stimulated gene expression that induced thermotolerance. Our results provide useful information for rice breeding and heat stress tolerance programs to benefit long-term global food security.

## 1. Introduction

The global climate is undergoing drastic changes with increasing temperatures and emissions of greenhouse gasses. Climate change is now threatening agriculture in all areas of the world, including Thailand. Average temperatures have increased since 1981, causing water deficiency and limiting plant growth for agricultural products [1].

Rice (*Oryza sativa* L.) plays a key role in global food security. Over 90% of rice is grown and consumed in Asian countries, especially China and India, while rice consumption is increasing in Latin America, Africa and the Caribbean. In Asia, rice is the major source of nutrition and a critical dietary crop for food security [2,3]. Global rice prices are predicted to increase with falling production due to climate change [4]. Rice production decreases by 8–10% for each 1 °C increase in temperature, while the average daily mean temperature has increased by 0.7 °C from 2009 to 2018 [5,6]. The optimal temperature range for rice growth is 23–33 °C, while the critical temperature for rice seedlings is 35 °C, as the highest temperature that allows proper growth. Higher temperatures have excessively detrimental consequences [7], and the average warmest temperature in Thailand from 2019–2021 was 42 °C [8].

Excessively high temperatures cause heat stress in plants, which impacts plant physiology, biochemical pathways, enzymatic reactions and transcriptomics. Heat stress induces lower stomatal conductance, with reduced CO_2_ assimilation and water loss. Lower CO_2_ concentration in leaves results in lower photosynthesis and higher photorespiration, which leads to reduced rice biomass, rice root elongation and decreased production [7]. Heat stress also directly affects other cell compartments with early effects, such as alteration of enzyme and protein structure and cell membrane permeability, resulting in photochemical modification in chloroplast, damage to the thylakoid membrane and inhibition of key enzymes in photosynthetic pathways, such as 1,5-bisphosphate carboxylase or Rubisco [9]. Heat stress also negatively affects rice reproductive stages, with panicle weight loss, reduced number of differentiated spikelets, spikelet fertility reduction and decreased grain weight [10]. Previous research concluded that heat stress hampers rice growth and development at every stage, with reduction in global rice production. Therefore, novel and effective strategies are required to breed heat stress-resistant rice cultivars.

KDML105 or Thai jasmine rice is renowned in the global market due to its outstanding features; the grains become soft and emit a fragrant perfume after cooking [11]. Physiological alteration is the major concern when rice encounters heat stress. Heat-exposed (40 °C) KDML105 seeds gave the highest percentage of abnormal seedlings, H_2_O_2_ accumulation and higher MDA content [12]. KDML105 rice seedlings exposed to heat stress showed significantly lower lipid saturation and long chain fatty acids, which were responsible for loss in membrane stability [13]. In addition to physiological changes, anatomical characteristics are also impacted by heat stress. Reduction in cell size and stomatal density and improvement of trichome were reported in rice exposed to heat stress [14]. The physiological response of rice to heat stress has been extensively documented; however, a comprehensive understanding of anatomical alteration and its integration with physiological and biochemical aspects is still limited, especially in high productivity rice, such as KDML105. Therefore, this study assessed the physiological and anatomical responses of KDML105 to heat stress and investigated the effects of heat stress on callus induction from KDML105 seeds to further advance heat stress tolerance in rice cultivars.

## 2. Materials and Methods

### 2.1. Plant Materials and Stress Conditions

KDML105 seeds were dehusked and sterilized with 15% sodium hypochlorite (Clorox) fortified with 2 drops of Tween 20 for 30 min with continuous agitation. Seeds were then rinsed at least three times with sterile distilled water before culture on MS solid medium for seed germination and MS solid medium supplemented with 3 mg/L 2,4-D for callus induction. MS medium was solidified with 8 g/L agar powder and the pH was adjusted to 5.8 before sterilization by autoclaving for 20 min at 121 °C.

Cultures of seed germination and callus induction experiments were exposed to three different stress conditions. The first was the control treatment with cultures incubated under 25 °C, while the stress condition was 35 °C. Both conditions were maintained at 16/8 h light/dark providing 40 µmol·m^−2^·s^−1^ for 4 weeks. Heat shock treatment was conducted under 42 °C for 1 week before further incubation under 25 °C for 3 weeks. After 4 weeks, shoot length, root length, number of leaves, fresh and dry weight, leaf greenness index by SPAD, MDA content, electrolyte leakage, chlorophyll content and anatomical characteristics were recorded for seed germination and response of seedlings to heat stress. Callus morphology as width and length and fresh and dry weight were collected under the control and stress conditions.

### 2.2. MDA Content

MDA content determination was carried out according to Velikova et al. [15] with some modifications. Fresh leaves (0.5 g) were ground with a mortar and pestle in 5 mL 0.1% trichloroacetic acid (TCA). The extracts were centrifuged at 14,000 rpm for 5 min. Supernatants were collected and 1 mL of supernatant was added with 4.5 mL of 0.5% thiobarbituric acid (TBA). The mixtures were boiled in a water bath for 30 min before cooling on ice. Absorbance was detected with three replications at 532 and 600 nm. MDA contents were calculated by the following equation:

MDA (µmole g FW-1) = ((A532 − A600) × Vf × Ve)/((155 × Va × FW))(1)

where

Vf = final volume
Ve = volume of TCA used for extraction
Va = volume of supernatants used in absorbance detection
FW = fresh weight of samples

### 2.3. Electrolyte Leakage

To determine electrolyte leakage, leaf samples (0.1 g) were immersed in 10 mL of deionized sterile water in test tubes and maintained under dark condition at 25 °C for one day. The initial electrical conductivity (EC1) was evaluated. The test tubes were then incubated at 100 °C for 15 min and cooled at 25 °C before the final electrical conductivity (EC2) was measured. Electrolyte leakages were calculated using the following equation:

Electrolytic leakage = EC1/(EC2) × 100(2)

### 2.4. Chlorophyll Content

Total chlorophyll was extracted from fresh leaves (25 mg) with 5 mL of 80% acetone under dark condition at 25 °C for two days and absorbance was measured with three replications at 645 and 663 nm. Total chlorophyll, chlorophyll a and chlorophyll b contents were calculated using the following equations:

(3) $$\text{Chlorophyll a (mg/g tissue)}=\frac{\left(12.7A663-2.69A645\right)\times V}{\left(1000\times W\right)}$$

(4) $$\text{Chlorophyll b (mg/g tissue)}=\frac{\left(22.9A645-4.68A663\right)\times V}{\left(1000\times W\right)}$$

(5) $$\text{Total chlorophyll (mg/g tissue)}=\frac{\left(20.2A645+8.02A663\right)\times V}{\left(1000\times W\right)}$$

where

V = volume of 80% acetone
W = sample fresh weight

### 2.5. Peeling Technique

Fresh leaves were immersed in 15% sodium hypochlorite for 10–15 min to macerate the leaf tissues. The adaxial side was peeled to remove the upper epidermis and mesophyll and the samples were washed before staining with 1% safranin O in water. The stained samples were observed under a microscope. Stomatal density, stomatal size and epidermal cell size were recorded.

### 2.6. Statistical Analysis

The experiment was conducted as a completely randomized design (CRD) with five replicates. Data were analyzed using one-way analysis of variance (ANOVA) at *p*-value = 0.05. Means were compared and separated by Duncan’s multiple range test (DMRT) using SPSS version 23.0. Pearson’s correlation and principal component analysis (PCA) were used to analyze and determine the relationships between growth and physiological parameters. Hierarchical cluster analysis (HCA) with a heatmap was used to group KDML105 rice seedlings cultured under different conditions based on growth and physiological data. Pearson’s correlation, PCA and HCA were conducted using Origin 2022 software.

## 3. Results

### 3.1. Seedling Growth and Development

**Figure 1:**
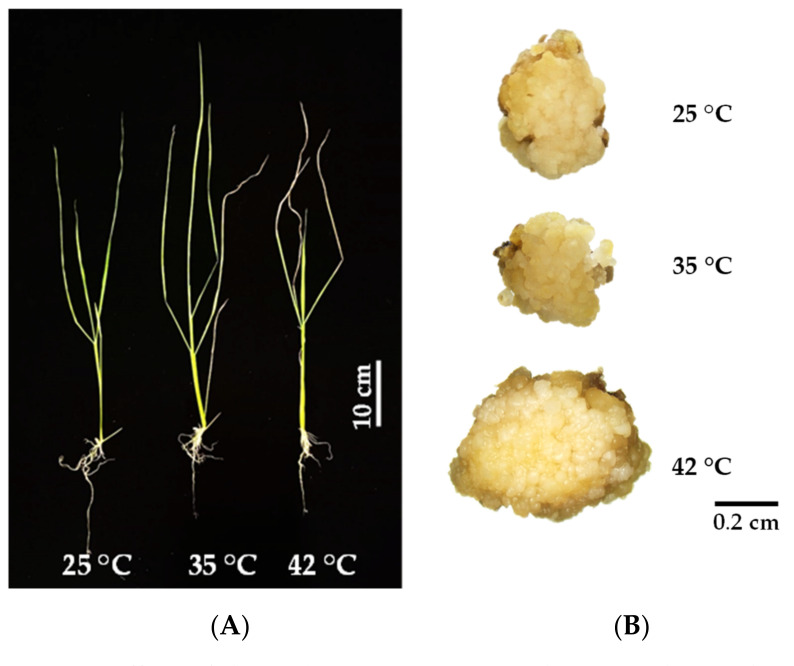
Effects of elevated temperatures (35 and 42 °C) and normal temperature (25 °C) on growth of KDML105 seedlings (**A**) and callus induction from KDML105 seeds (**B**).

Survival percentages of KDML105 rice seedlings among the control and stress conditions were not impacted by heat stress. However, heat stress had a strong adverse influence on their development, with seedling growth reduced at elevated temperatures (Table 1; Figure 1A; Supplementary file). Shoot and root length of rice seedlings in the control (27.20 and 6.16 cm) and 35 °C (26.39 and 6.29 cm) treatments were significantly higher than rice seedlings grown under 42 °C (24.09 and 4.53 cm; *p* < 0.05). Negative effects of heat stress were also found in fresh and dry weight of rice seedlings, with the control (25 °C) at 373.84 and 60.75 mg and significantly higher than 35 °C, 172.42 and 32.08 mg, and 42 °C, 189.12 and 41.50 mg (*p* < 0.05). Results indicated that elevated temperatures were the major cause of seedling growth impairment. Rice seedlings from the control treatment provided a largest number of six leaves, significantly higher than the stress treatments (4.25 leaves at 35 °C and 4.75 leaves at 42 °C) (*p* < 0.05). Heat stress had no effect on leaf length, but leaf width reduced in seedlings grown at 35 and 42 °C (0.19 and 0.21 cm), compared to the control (0.27 cm; *p* < 0.05).

### 3.2. Callus Induction of KDML105 under Heat Stress

In addition to growth, physiological and anatomical alteration due to heat stress, this study also focused on the effects of elevated temperature on callus induction from KDML105 seeds (Figure 1B). Results showed the negative effects of heat stress on callus survival. KDML105 calli grown at 35 and 42 °C gave 66.67% callus survival, lower than the control (25 °C) but with no significance (Table 1; *p* > 0.05). However, callus size and weight did not exhibit similar results. The widest callus was observed at 42 °C, while the longest was found in culture at 35 °C. Calli induced by heat shock at 42 °C provided the highest fresh and dry weight. Differences among treatments were not significant (*p* > 0.05) but size and weight of KDML105 calli increased when cultured under heat stress.

### 3.3. Physiological Response of KDML105 Rice Seedlings to Heat Stress

In this study, heat stress affects KDML105 rice seedling growth and development and also hinders seedling physiology. Chlorophyll degradation resulted from heat stress because elevated temperatures (35 and 42 °C) induced a significant reduction in total chlorophyll content (0.19 and 0.18 mg/g tissue), chlorophyll a (0.13 and 0.12 mg/g tissue) and chlorophyll b (0.06 and 0.06 mg/g tissue), compared to the control temperature (*p* < 0.05; Table 1). These results were consistent with the SPAD values. Seedlings from the control treatment (29.17 SPAD units) provided the significantly highest SPAD value, while values reduced under heat stress (25.00 SPAD units at 35 °C and 27.83 SPAD units at 42 °C) (Table 1). Percentages of electrolyte leakage at 35 and 42 °C were 40.25% and 44.71%, respectively, and increased compared to the control treatment at 28.24% (*p* < 0.05; Table 1).

### 3.4. Pearson’s Correlation, Principal Component Analysis (PCA) and Hierarchical Cluster Analysis (HCA) for Growth and Physiological Parameters

**Figure 2:**
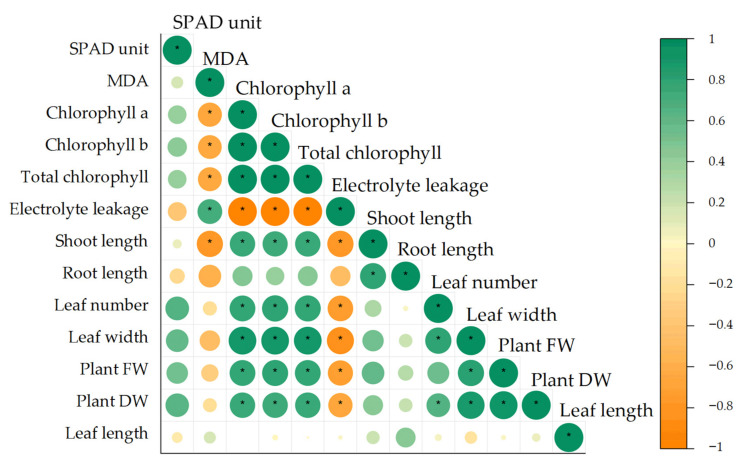
Pearson’s correlation among growth and physiological parameters of KDML105 seedlings to heat stress (asterisk, *p* < 0.05).

The relationships between growth and physiological data were determined using Pearson’s correlation and represented as a biplot of principal component (PC) 1 and PC2 using principal component analysis. Results showed a positive strong correlation between MDA content and electrolyte leakage. Positive correlations were also found among other physiological (chlorophyll a, b and total chlorophyll) and growth (shoot length, root length, leaf number, leaf width, fresh and dry weight) parameters, while MDA and electrolyte leakage negatively correlated with these parameters. SPAD unit and leaf length were not significantly correlated with the other parameters (Figure 2).

PCA was then analyzed from correlation coefficients and the biplot was represented. Data variances were explained by the eight principal components at 100%. The first two components, PC1 and PC2 explained data variation for 63.57% and 17.84%, respectively, and were selected to create a PCA biplot which explained 81.41% of the variation. PC1 was mainly composed of MDA content, chlorophyll a, chlorophyll b, total chlorophyll content, electrolyte leakage, shoot length, leaf number, leaf width, fresh weight and dry weight, while PC2 explained SPAD unit and root length (Table 2).

**Figure 3:**
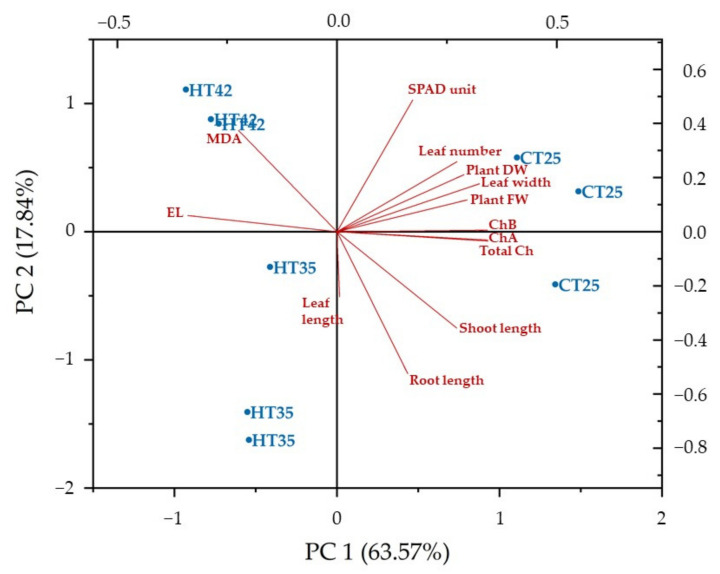
PCA biplot for PC1 and PC2 representing the relationships between KDML105 seedlings and growth and physiological parameters recorded from the control and heat stress conditions.

The PCA biplot showed that MDA content and electrolyte leakage were negatively correlated, and both parameters increased in KDML105 seedlings due to heat stress, while the other parameters positively correlated with the PC1 axis. The acute angle between MDA content and electrolyte leakage confirmed their positive correlation, while relationships among chlorophyll contents and growth parameters were also confirmed by acute angles. However, the biplot also showed that MDA content and electrolyte leakage had negative correlations with the other parameters, as indicated by the obtuse angles between them (Figure 3).

**Figure 4:**
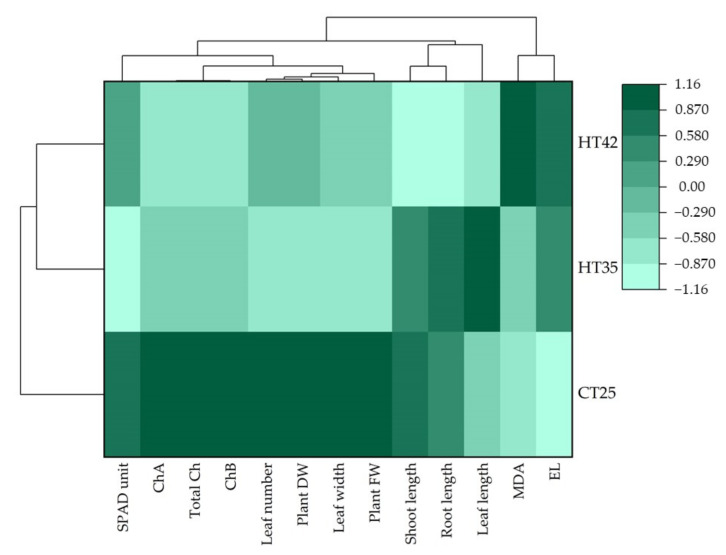
Hierarchical cluster analysis and heatmap explaining the responses of KDML105 seedlings on heat stress at different levels (25, 35 and 42 °C) (ChA = chlorophyll a; ChB = chlorophyll b; DW = dry weight; FW = fresh weight; MDA = malondialdehyde; EL = electrolyte leakage).

Hierarchical cluster analysis (HCA) and a heatmap were analyzed to explain the overview that KDML105 seedling clusters depended on growth and physiological responses due to heat stress. The HCA results were consistent with PCA analysis. Three clusters of parameters were identified. Cluster I consisted of MDA content and electrolyte leakage. Cluster II contained leaf length, shoot and root length, chlorophyll and growth parameters, while chlorophyll a, chlorophyll b, total chlorophyll content, leaf number, leaf width and fresh and dry weight belonged to cluster III (Figure 4).

### 3.5. Leaf Surface Anatomy of KDML105 Rice Seedlings Grown under Heat Stress

**Figure 5:**
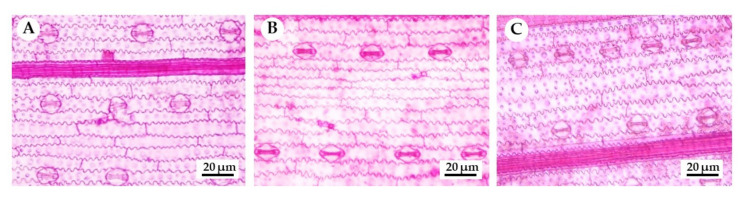
Surface anatomy of KDML105 leaves at different levels of heat stress. (**A**) The control or 25 °C; (**B**) 35 °C; (**C**) 42 °C.

Growth and physiology of KDML105 rice seedlings were influenced by heat stress, as mentioned above. Anatomical changes were further investigated to clarify the adaptation of KDML105 rice seedlings grown under elevated temperature (Figure 5). Results demonstrated that stomatal length of seedlings grown under 35 °C (13.35 µm) was significantly higher than at the other temperatures (*p* < 0.05), while significant differences were not observed in stomatal width (Table 3). Stomatal density increased at higher temperature but there was no significant difference among the treatments (*p* > 0.05). A similar trend was found in short- and long-epidermal cell length (Supplementary file). Seedlings from the 35 °C treatment gave the widest epidermal cells in both types but differences were not significant (Table 3; *p* > 0.05).

## 4. Discussion

This study investigated the responses of KDML105 seedlings to high temperature in different aspects. Obvious impacts were shown by growth and development. Elevated temperature due to global warming is detrimental to crop plants, especially for root growth at the seedling stage. Results showed that elevated temperature in the culture chamber had a detrimental effect on shoot and root length, leaf characteristics and biomass of KDML105 seedlings, especially the treatment at 42 °C (Figure 1A; Table 1). Negative effects on plant growth were also observed in other plant species. The seedlings of fifteen alfalfa phenotypes treated with heat stress for a week showed reduced shoot and root weight [16]. Short time heat stress also hinders seedling growth. Shoot and root weights of chili pepper seedlings decreased after heat stress treatment for 4–12 h [17]. Loss of biomass was also observed in rice seedlings. A short period as 24 h of heat stress induced a slightly higher percentage of seed germination and shoot length in rice var. Kitaake but exposure to heat stress for 72 h showed significantly lower parameter values [18]. Prasertthai et al. [13] found that KDML105 rice seedlings showed a reduction in most growth parameters when they were cultivated under heat stress. Heat stress also induced abnormalities, such as white and shortened roots and yellow or brown curving leaves in KDML105 and riceberry seedlings grown under higher temperature [12]. This phenomenon was explained by water limitation and reduced mineral uptake. Small increases in temperature induced higher stomatal conductance, particularly in stress-tolerant species but stomatal conductance in heat susceptible wheats decreased under heat stress [19].

Slightly higher temperatures or short-term heat stress caused higher stomatal conductance, while excessive heat stress induced decreased stomatal conductance, resulting in lower water uptake by the roots and lower nutrient absorption, especially nitrogen [20]. Giri et al. [21] found that heat stress impacted tomato seedlings, especially roots with reduction in root mass. They also found that short-term heat stress increased stomatal conductance to promote leaf surface cooling but this significantly decreased at prolonged heat stress, with decreased nitrogen and carbon accumulation in tomato roots. Stomatal conductance was also related to photosynthesis efficiency [22]. Consequently, growth and development of KDML105 were interrupted by heat stress through lower stomatal conductance, leading to lower nutrient and water uptake and alteration of seedling physiology.

Our findings showed that physiological responses were also negatively impacted by heat stress. Elevated temperatures cause stresses to plant cell compartments, including the cell membrane [13]. MDA content and electrolyte leakage parameters that indicate membrane stability [12] were evaluated to determine cell membrane damage due to heat stress. Results demonstrated that MDA content in KDML105 rice seedlings grown under 42 °C (0.3 µmole mg FW^−1^) was significantly higher than in seedlings from the control (0.02 µmole mg FW^−1^) and 35 °C (0.09 µmole mg FW^−1^) conditions (*p* < 0.05; Table 1).

With increasing MDA content and electrolyte leakage, chlorophyll a, chlorophyll b and total chlorophyll content of KDML105 seedlings drastically decreased (Table 1). Chloroplast is an organelle responsible for photosynthesis that contains photosystem (PS) on the thylakoid membrane. Thylakoid grana and membrane are very sensitive to heat stress because they are always degraded when plants are exposed to elevated temperature, eventually resulting in decreased photosynthesis [23]. Heat-tolerant species showed constant chlorophyll content or even a slight decrease in photosynthesis efficiency, while heat stress-sensitive species presented decreasing chlorophyll content or photosynthesis [13,24]. Sánchez-Reinoso et al. [25] found that rice cultivar F473 seedlings treated at 35 and 40 °C gave decreased chlorophyll a, chlorophyll b and total chlorophyll content, with lower maximum quantum yield of PSII efficiency (*F_v_/F_m_*). Decreased chlorophyll a and chlorophyll b content was also found in wheat cultivars JM22 and XM26 after heat stress for 5 and 10 days, with lower *F_v_/F_m_* [24]. Results also revealed that heat stress caused chlorophyll degradation in KDML105 rice seedlings, leading to lower photosynthesis.

Reactive oxygen species (ROS) are produced when plants encounter different stresses, including heat stress [13]. The major source of ROS is localized in chloroplasts through electron transport chains in PSI and PSII. The overaccumulation of ROS damages thylakoids and disturbs cellular activities in chloroplasts [26]. Oxidative stress due to ROS overaccumulation impairs cell membranes by lipid peroxidation, leading to MDA accumulation. Damages from heat stress also alter the membrane structure, resulting in electrolyte leakage from inside plant cells [11]. MDA and electrolyte leakage are used to determine cell membrane integrity after plants encounter different stresses [23]. Increasing MDA content and electrolyte leakage due to heat stress were observed in several plants. Tomato leaves of thermosensitive and thermotolerant genotypes exposed to heat stress showed elevated MDA content and electrolyte leakage [27]. Electrolyte leakage of Indian mustards increased in both thermotolerant and thermosensitive genotypes ranging from 2–2.75-fold after heat stress treatment, compared to the control, while MDA content was notably higher in thermosensitive genotypes [28]. Physiological alterations resulting from heat stress were also noticed in rice. Three Colombian rice cultivars showed higher MDA content and electrolyte leakage when seedlings were treated with heat stress [25]. ROS from oxidative stress react with unsaturated lipids contained in the membranes and produce toxic aldehydes, including MDA. High MDA content then induces membrane damage from lipid peroxidation and impacts photosynthesis efficiency [29]. Heat stress also induces alterations in membrane fluidity, permeability and lipid compositions, causing membrane instability through electrolyte leakage [13,30]. Our results concurred with previous studies that KDML105 rice seedlings had significantly higher MDA content and electrolyte leakage under heat stress than the control seedlings, indicating that KDML105 rice is sensitive to heat stress [13].

As previously discussed, abiotic stresses, including heat stress cause ROS overaccumulation and lead to programmed cell death [31]. ROS are always accompanied by positively correlated electrolyte leakage and lipid peroxidation [32]. A study in tobacco under drought stress revealed that MDA content and ion leakage showed strong negative correlation with chlorophyll content and plant biomass [33], similar to observations between H_2_O_2_ and other growth parameters in citrus rootstocks under drought stress [34]. ROS accumulated during heat stress led to impaired growth and development of KDML105 seedlings.

Heat stress impacted on the growth and physiology of KDML105 seedlings. Correlation between growth and physiological parameters was further analyzed to reveal the association in response to heat stress. PCA results also demonstrated diverse growth and physiological parameter responses of KDML105 seedlings to heat stress. The biplot (Figure 3) shows KDML105 seedlings without heat stress grouped into the two right quadrants associated with growth and chlorophyll parameters, indicating that under normal conditions seedlings grow well with high photosynthesis efficiency due to the stability of chloroplasts [35], with lower MDA content and electrolyte leakage. Similar results in citrus rootstocks and melon plants suggested that plants are not impacted by drought and salt stress was positively associated with chlorophyll, growth and photosynthesis parameters [34,36]. Heat-treated KDML105 seedlings were separated into two subgroups associated with MDA content and electrolyte leakage. Seedlings grown under 35 °C were grouped in the lower left quadrant, while seedlings grown under 42 °C were grouped in the upper left quadrant and strongly associated with MDA content and electrolyte leakage. These two parameters were related to membrane stability because MDA is a byproduct of lipid peroxidation due to ROS accumulation, while membrane compositions were altered by heat stress [13,30]. These reasons caused instability and damage to the thylakoid membrane composition resulting in chlorophyll degradation and decline in photosynthesis efficiency [37]. Results proved that MDA content and electrolyte leakage, as factors for membrane instability, were responsible for heat stress in KDML105 seedlings. A previous study on *Phyllanthus amarus* treated with drought stress for 10 days found an association with lipid peroxidation [38]. Therefore, MDA content and electrolyte leakage are effective indicators of heat stress in plants.

HCA results also showed that the responses of KDML105 seedlings to heat stress treatments at the three levels were different. Seedlings from the control condition (25 °C) showed high levels of parameters in clusters II and III, whereas a decrease in cluster I was observed. When seedlings were cultured under 35 °C, a decline in cluster II was noticed along with an increase in cluster I, suggesting that heat stress at 35 °C caused impairment in seedling growth and elevated levels of lipid peroxidation and membrane instability. However, no significant alteration was observed in cluster II. Severe changes in response to heat stress were observed in seedlings grown at 42 °C. Clusters II and III levels decreased, while MDA content and electrolyte leakage (cluster I) drastically increased, demonstrating that KDML105 seedlings suffered from lipid peroxidation due to ROS and membrane instability that led to chlorophyll degradation, growth impairment and eventual plant death. The HCA results confirmed the PCA biplot and explained the growth and physiological responses of KDML105 seedlings under heat stress.

A decrease in stomatal conductance is one of the adaptation mechanisms that plants use to survive under higher temperature [21,39]. In addition to physiological alterations, plants also undergo anatomical adaptations, especially in stomata numbers on the leaf surface when grown under high environmental temperature. Shen et al. [40] revealed that higher environmental temperature increased stomatal density because stomata were newly formed to relieve cell and tissue damage, resulting from heat stress. Our anatomical results showed a slight increase in stomatal density of KDML105 rice seedlings grown at 42 °C (Table 3, Figure 5). However, no significant difference in stomatal density was found and stomatal size did not alter among the treatments, concurring with several previous studies. Stomatal density and size on both the abaxial and adaxial epidermis of soybean leaves grown under heat stress were not significantly different to the control [41]. Zhou et al. [42] found no obvious effects of heat stress on stomatal size and density of tomato ‘Aromata’. Generally, plants that encounter heat stress possess higher stomatal density and smaller stomata to improve heat resistance efficiency. Reduced leaf area induced by heat stress results in higher stomatal density which is not an anatomical adaptive mechanism [43]. These results suggested that plants do not rely on increased stomatal density to overcome heat stress and apply other mechanisms, including an increase in stomatal pore size or number of stomatal pore openings [42], which develop higher stomatal conductance and photosynthesis efficiency. Leaf surface anatomy of KDML105 seedlings did not show a significant response to heat stress but other anatomical characteristics, such as leaf and stem anatomy, were adversely influenced. The anatomical response of KDML105 rice to heat stress requires further study to clarify this finding.

This study reported on growth, physiological and anatomical responses to heat stress while impact on callus induction was also investigated. Some studies exhibited the detrimental effects of heat stress on callus induction efficiency. Calli of two wheat genotypes demonstrated decreasing callus area and callus proliferation efficiency when culture temperature increased [44], consistent with our results that callus survival percentages in KDML105 decreased as culture temperature increased. Heat stress may not impact photosynthesis and stomatal conductance of callus as much as mature plants. Oxidative stress and excessive accumulation of ROS alter redox homeostasis and lead to membrane instability [45,46]. Notably, this study provided contradictory results, demonstrating that KDML105 callus size and weight increased but without significant differences when treated under heat stress. A similar phenomenon has been noticed in several plant species. Seeds of four Malaysian rice cultivars pretreated with heat stress, 35–50 °C, showed that calli were initiated from seeds earlier than non-pretreated seeds. Seeds pretreated at 45 °C provided highest calli fresh and dry weight [47]. The callus induction of moth bean was accelerated from 13 days of callus initiation in the control to 11 days after heat shock treatments for 10 min [48]. Plant regeneration was also positively affected by heat stress. Pretreatment of calli at 35 °C for 6 h provided the highest percentage of plant regeneration in barley [49]. Heat stress stimulates protein accumulation, phytohormones and gene expression related to carbohydrate, lipid and protein metabolism, antioxidant enzymes and ROS scavenging [50], while proteins and phytohormones induce thermotolerance in plants [51].

## 5. Conclusions

This study focused on the physiological and anatomical responses of KDML105 to heat stress and the relationships among these parameters. Results demonstrated that heat stress induced growth and physiological alterations. Chlorophyll contents, growth and biomass of KDML105 seedlings were maintained under the control condition, with low levels of MDA and electrolyte leakage observed. By contrast, KDML105 seedlings grown under heat stress showed impaired growth and chlorophyll degradation. MDA content and electrolyte leakage levels also increased due to excessive ROS accumulation. The PCA and HCA results also confirmed negative correlations between MDA content, electrolyte leakage and other parameters. MDA content and electrolyte leakage were shown to be effective indicators of heat stress in rice. Surface anatomical responses of seedlings to heat stress were not observed, indicating that other mechanisms were preferred by KDML105 seedlings to deal with heat stress. Heat stress on callus induction from KDML105 seeds showed no significant negative effects, while mass of calli increased because heat stress stimulated protein accumulation, phytohormones and gene expressions that induced thermotolerance. Our results provide useful information for rice breeding and heat stress tolerance programs to benefit long-term global food security.

## Figures and Tables

**Table 1:** Growth, physiological and anatomical responses of KDML105 seedlings and callus induction efficiency from KDML105 seeds after treatment at 25 (control), 35 and 40 °C.

Characteristics	Temperatures (°C)		
	25	35	42
**Growth of seedlings**			
Survival percentage (%)	100.00 ± 0.00 a	100.00 ± 0.00 a	100.00 ± 0.00 a
Shoot length (cm)	27.20 ± 0.41 a	26.39 ± 0.18 a	24.09 ± 0.09 b
Root length (cm)	6.16 ± 0.64 a	6.29 ± 0.26 a	4.53 ± 0.33 b
Leaf number	6.00 ± 0.38 a	4.25 ± 0.14 b	4.75 ± 0.25 b
Leaf width (cm)	0.27 ± 0.00 a	0.19 ± 0.00 b	0.21 ± 0.00 c
Leaf length (cm)	14.93 ± 1.20 a	16.57 ± 0.28 a	14.35 ± 3.70 a
Fresh weight (mg)	373.84 ± 75.13 a	172.42 ± 16.63 b	189.12 ± 19.91 b
Dry weight (mg)	60.75 ± 7.01 a	32.08 ± 2.42 b	41.50 ± 3.19 b
**Physiological characteristics**			
SPAD value	29.17 ± 0.02 a	25.00 ± 2.00 b	27.83 ± 0.07 ab
MDA (µmole mg FW^−1^)	0.02 ± 0.00 b	0.09 ± 0.04 b	0.30 ± 0.07 a
Electrolyte leakage (%)	28.24 ± 0.56 c	40.25 ± 0.27 b	44.71 ± 0.10 a
Chlorophyll a (mg/g tissue)	0.1812 ± 0.0001 a	0.1305 ± 0.0021 b	0.1194 ± 0.0016 c
Chlorophyll b (mg/g tissue)	0.0780 ± 0.0013 a	0.0614 ± 0.0001 b	0.0583 ± 0.0003 c
Total chlorophyll (mg/g tissue)	0.2592 ± 0.0011 a	0.1919 ± 0.0035 b	0.1776 ± 0.0031 c
**Callus induction**			
Survival percentage (%)	91.67 ± 8.33 a	66.67 ± 8.33 a	66.67 ± 8.33 a
Callus width (cm)	0.34 ± 0.05 a	0.46 ± 0.02 a	0.54 ± 0.13 a
Callus length (cm)	0.64 ± 0.09 a	0.96 ± 0.25 a	0.80 ± 0.17 a
Fresh weight (mg)	31.80 ± 0.01 a	54.80 ± 0.01 a	73.2 ± 0.09 a
Dry weight (mg)	7.00 ± 0.00 a	10.8 ± 0.00 a	13.2 ± 0.01 a

Means ± SE followed by different letters are significantly different in rows by one-way ANOVA and Duncan’s multiple range test (DMRT; *p* < 0.05).

**Table 2:** Loading variables, variance, cumulative variance and eigenvalues from PCA analysis of growth and physiological parameters after heat stress of KDML105 seedlings.

Parameter	PC1	PC2	PC3	PC4	PC5	PC6	PC7	PC8
SPAD unit	0.495	0.742	0.171	0.050	0.413	0.033	−0.034	−0.010
MDA	−0.645	0.571	0.470	0.023	−0.019	0.088	0.171	−0.006
Chlorophyll a	0.988	−0.053	−0.056	−0.093	−0.056	0.036	0.040	−0.047
Chlorophyll b	0.985	0.008	−0.084	−0.107	−0.042	−0.071	0.065	−0.004
Total chlorophyll	0.986	−0.047	−0.047	−0.103	−0.078	0.012	0.058	−0.049
Electrolyte leakage	−0.977	0.091	0.106	0.133	−0.067	0.031	−0.043	0.010
Shoot length	0.783	−0.542	0.028	0.125	0.257	−0.106	−0.007	0.008
Root length	0.462	−0.799	0.246	0.096	0.080	0.256	0.071	0.027
Leaf number	0.785	0.395	0.114	−0.460	−0.035	0.007	0.006	0.052
Leaf width	0.932	0.269	−0.101	0.039	−0.112	0.144	−0.108	0.049
Leaf length	0.852	0.179	0.163	0.405	−0.111	−0.167	0.089	0.049
Plant DW	0.831	0.321	0.266	0.314	−0.143	0.075	−0.099	−0.044
Plant FW	0.017	−0.367	0.899	−0.192	−0.048	−0.103	−0.085	−0.007
Variance (%)	63.570	17.844	9.718	4.530	2.365	1.217	0.640	0.117
CV (%)	63.570	81.415	91.132	95.662	98.027	99.243	99.883	100.000
Eigenvalue	8.264	2.320	1.263	0.589	0.307	0.158	0.083	0.015

Abbreviation: PC, principal component; MDA, malondialdehyde; CV, cumulative variance DW, dry weight; FW, fresh weight.

**Table 3:** Anatomical responses of KDML105 seedlings after treatment at 25 (control), 35 and 40 °C.

Characteristics	Temperatures (°C)		
	25	35	42
Stomatal length (µm)	11.24 ± 0.19 b	13.35 ± 0.55 a	11.76 ± 0.41 b
Stomatal width (µm)	8.97 ± 0.15 a	9.93 ± 0.45 a	8.88 ± 0.19 a
Stomatal density	37.00 ± 0.58 a	34.33 ± 1.20 a	39.33 ± 3.84 a
Short-epidermal cell length (µm)	25.13 ± 0.93 a	28.35 ± 1.02 a	28.44 ± 1.64 a
Short-epidermal cell width (µm)	5.95 ± 0.00 a	6.83 ± 0.37 a	5.82 ± 0.90 a
Long-epidermal cell length (µm)	59.61 ± 3.37 a	61.15 ± 4.99 a	72.44 ± 4.76 a
Long-epidermal cell width (µm)	6.75 ± 0.20 a	7.19 ± 0.19 a	6.61 ± 0.40 a

Means ± SE followed by different letters are significantly different in rows by one-way ANOVA and Duncan’s multiple range test (DMRT; *p* < 0.05).

## Data Availability

The raw data can be found in the Supplementary file.

## Supplementary Materials

The following supporting information can be downloaded at: https://www.mdpi.com/article/10.3390/biology11111587/s1.
